# Commentary: Seeing the conflict: an attentional account of reasoning errors

**DOI:** 10.3389/fpsyg.2017.01284

**Published:** 2017-07-25

**Authors:** Darren P. Frey, Bence Bago, Wim De Neys

**Affiliations:** ^1^Université Paris Descartes Paris, France; ^2^University of Caen Normandy Caen, France; ^3^UMR8240 Laboratoire de Psychologie du Développement et de l'Education de L'enfant Paris, France

**Keywords:** intuition, reasoning, decision making, conflict detection, attention

In a recent study, Mata et al. (2017) test a two-stage account of reasoning that differentiates between the initial interpretation and selection of information within a problem, and the subsequent operation on this information. Crucially, on this account, reasoning errors can result from mistakes at either stage. In previous work, the authors found indirect experimental evidence in support of this account (e.g., Mata et al., 2014), and their most recent experiments address it by analyzing reasoners' on-line attentional resources in situations of conflict. To do so, the authors record eye-tracking measurements as participants evaluate both classical reasoning tasks that induce conflict (variants of the bat-and-ball item from the Cognitive Reflection Test, Frederick, 2005), and comparably structured no-conflict items. Their two-stage account makes at least two strong predictions. All things being equal, correct responders should: (1) attend to conflict problems more than no-conflict items; (2) and they should attend to conflict items more than incorrect responders.

Mata et al. present two studies, the second of which is a replication of the first that avoided a potential confound present in Study 1. In that study, eye-tracking recordings ended when participants indicated that they were ready to respond, raising the concern that crucial aspects of the reasoning process might have been neglected (Ball et al., 2006). By and large, the results of their studies support the claim that correct responders attend more to conflict than no-conflict items, and they allocate more attentional resources to the critical components of the tricky conflict items. Using a technique that is fairly novel in this context (e.g., see Ball et al., 2006, for eye-tracking studies with a different reasoning task; see Ball, 2013 for a review), the authors have generated interesting insights into how it is that reasoners may be led to err, suggesting a largely overlooked attentional path to such mistakes.

However, the authors further interpret their results to indicate that incorrect responders are generally insensitive to conflicts. It is one thing to assert that correct responders do a better job at attending to conflicts than incorrect responders, but it is quite another to claim that incorrect responders are entirely insensitive to such conflicts. In line with Mata et al.'s findings, there is previous experimental literature that suggests that correct responders are more sensitive to conflicts than incorrect responders (e.g., Pennycook et al., 2015). Among incorrect responders, there are prominent individual differences. Within samples of biased responders there are subgroups of participants across various tasks who do, indeed, fail to detect reasoning conflicts. This group tends to range from 15 to 40% of incorrect responders (Mevel et al., 2015; Pennycook et al., 2015; Frey et al., 2017). Yet, the majority of even biased individuals detect conflict across a number of tasks that make use of different measures, suggesting that there is no good empirical reason to universally equate incorrect responding with insensitivity to conflict. A great deal of evidence suggests that incorrect responders are often at least minimally sensitive to conflicts across a wide range of tasks. Surveying this literature, De Neys (2012, 2014) cites evidence of conflict sensitivity on a plurality of diverse tasks (base rate neglect, conjunction fallacy, ratio bias, syllogistic reasoning). More importantly, using the very same task as Mata et al. (2017)—the bat-and-ball problem—different teams have found evidence that incorrect responders detect conflicts on such items (De Neys et al., 2013; Gangemi et al., 2015; Johnson et al., 2016). Since the latter findings rely on different measures (reaction times and confidence/error ratings) than those employed by Mata et al. (2017), one might argue that these contradictory results are a function of the distinct techniques the experiments used. Perhaps the subtler, less invasive eye-tracking results are particularly well positioned to uncover misrepresentations of the conflict premises attributable to early attentional divergences. Moreover, there are dissenting accounts using different techniques. For instance, Travers et al. (2016) find no evidence of conflict detection among incorrect responders using mouse-movement tracking methods.

Because this is somewhat controversial territory, perhaps it is worth considering the actual data Mata et al. (2017) use to draw their conclusion. The authors base their claim for the insensitivity of incorrect responders to reasoning conflict on null results drawn from frequentist statistics in a small sample (*n* = ~30 in Study 1; and *n* = 27 in Study 2). The authors briefly note the possibility that their sample of incorrect responders might have been underpowered and therefore incapable of discerning differences in this group. To test this, we ran a Bayesian analysis on their data using Bayes factors. This approach allows us to quantify the degree of support for the null hypothesis (e.g., Wagenmakers, 2007; Masson, 2011; Morey et al., 2014).

Given the potential confound resulting from the non-continuous nature of the eye-tracking in Study 1, we focused on Study 2, and the authors were kind enough to share their subject-averaged results with us. Using the JASP package (JASP Team, 2016), we ran a Bayesian paired samples *t-*test with default priors (Cauchy prior width = 0.707). We entered the average fixation time and number of revisits on the critical problem premises (sentence 3) on conflict and no-conflict items for the group of incorrect responders in the analyses. Results showed that the Bayes factor in favor of the null hypothesis (no effect of conflict) over the alternative hypothesis (effect of conflict) was 1.34 for fixation time (non-directional; directional alternative hypothesis conflict > no-conflict, Bayes factor = 0.71) and 2.84 for the number of revisits (non-directional; directional alternative hypothesis conflict > no-conflict, Bayes factor = 1.67). These Bayes factor values indicate that there is only weak support in favor of the null hypothesis. Based on the classification of Lee and Wagenmakers (2013), the evidence for the hypothesis that incorrect responders do not distinguish between the conflict and no-conflict versions of the problem can be classified as merely anecdotal. Indeed, the only clear conclusion that we can draw based on these data is that we need more data to support a convincing conclusion in either direction.

In sum, although there is much to like about the Mata et al. (2017) paper, our key point is that the study does not present substantial evidence for the claim that incorrect responders cannot discriminate between conflict and no-conflict problems. Given the prior contradictory findings on the exact same task, we propose that caution is needed when drawing strong conclusions about incorrect responders' insensitivity to conflict on the basis of this study.

## Author contributions

DF wrote the article. BB and WD analyzed the data. WD revised the article.

### Conflict of interest statement

The authors declare that the research was conducted in the absence of any commercial or financial relationships that could be construed as a potential conflict of interest.
